# Complex Field Network Coding for Multi-Source Multi-Relay Single-Destination UAV Cooperative Surveillance Networks

**DOI:** 10.3390/s20061542

**Published:** 2020-03-11

**Authors:** Rui Xue, Lu Han, Huisi Chai

**Affiliations:** 1 College of Information & Communication Engineering, Harbin Engineering University, Harbin 150001, China; hanlu@hrbeu.edu.cn; 2 China Research Institute of Radiowave Propagation, Xinxiang 453000, China; chaihs@crirp.ac.cn

**Keywords:** unmanned aerial vehicle (UAV), cooperative communication, topology structure, complex field network coding (CFNC)

## Abstract

Relay-based cooperative communication for unmanned aerial vehicle (UAV) networks can obtain spatial diversity gains, expand coverage, and potentially increase the network capacity. A multi-source multi-relay single-destination structure is the main topology structure for UAV cooperative surveillance networks, which is similar to the structure of network coding (NC). Compared with conventional NC schemes, complex field network coding (CFNC) can achieve a higher throughput and is introduced to surveillance networks in this paper. According to whether there is a direct communication link between any source drone and the destination, the information transfer mechanism at the downlink is set to one of two modes, either mixed or relay transmission, and two corresponding irregular topology structures for CFNC-based networks are proposed. Theoretical analysis and simulation results with an additive white Gaussian noise (AWGN) channel show that the CFNC obtains a throughput as high as 1/2 symbol per source per channel use. Moreover, the CFNC applied to the proposed irregular structures under the two transmission modes can achieve better reliability due to full diversity gain as compared to that based on the regular structure. Moreover, the reliability of the CFNC scheme can continue to be improved by combining channel coding and modulation techniques at the expense of rate loss.

## 1. Introduction

**Figure 1:**
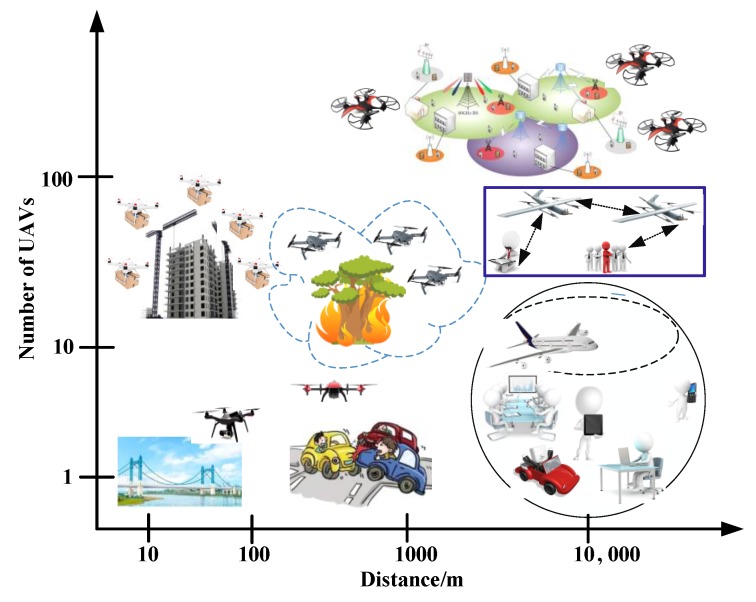
Application areas over a range of distance vs. number of nodes.

Recently, wireless communications aided by unmanned aerial vehicles (UAVs, also known as drones) have drawn a lot of attention from academic and industrial fields, as well as the general public [1]. Due to their ease of deployment, low cost, high mobility, and ability to hover [2] compared to conventional terrestrial infrastructure, UAVs hovering in the air are more likely to set up wireless links with favorable channel conditions and thus are considered as a promising vector of support for wireless communications in a great number of practical applications [3], such as security and surveillance, the real-time monitoring of road traffic, providing wireless coverage, remote sensing, search and rescue operations, the delivery of goods, precision agriculture, and civil infrastructure inspection [4]. However, it is difficult to complete the complex missions with a single UAV because of its limited detection capacity, energy resources, load, and other factors [5]. The solution to such a problem is ad-hoc formation using multiple UAVs [6]. The number of UAVs and their travel distances vary over a wide range for different applications here, as shown in Figure 1 [2]. Multiple small UAVs as a swarm to complete various tasks have gained more interest, as they improve the effectiveness of a single UAV system [7].

An emerging swarm application is the use of small UAVs as source nodes to collect information by their own airborne sensors, and the use of other UAVs as relay nodes to form reliable communication links for ad-hoc ground networks in tactical situations [8,9,10]. With the application of new sensors (e.g., high-definition aviation digital cameras, airborne imaging spectrometers, aviation imaging radars, etc.) in a single UAV, the information gathered from several source drones is sharply increased. Therefore, determining how to improve the throughput of UAV surveillance networks is a problem worth studying. A multi-source multi-relay single-destination (MSMRSD) structure is the main topology structure of UAV cooperative surveillance networks, and clusters are formed respectively among the source nodes and relay nodes. Effective information sharing among closely spaced intra-cluster nodes (i.e., among source nodes and/or among relay UAVs) is used to facilitate the cooperation [11], which is similar to the structure of network coding (NC) [12]. NC is an effective method to increase network throughput, and a real-time of UAV communication system can be greatly enhanced by introducing network coding principles.

NC is a technique used for effective and secure communication by improving network capacity, throughput, efficiency, and robustness [13]. Its core idea is to employ intermediate nodes to process the received data rather than the traditional forwarding of data, i.e., linear combination or some kind of coding to previously received information. The destination nodes can recover the original data by the part of received data, such that the throughput of the network is efficiently improved and the network’s security is increased [14]. Up to now, the main application of network coding in UAV communication networks has been random linear network coding (RLNC) [15,16] or physical-layer network coding (PNC) [17,18,19]. RLNC can achieve throughput arbitrarily close to the capacity in an unreliable single-hop broadcast network while yielding an acceptable decoding delay [20]. However, the throughput advantage of RLNC in a dynamic UAV network does not seem to be remarkable when the topology of a UAV network is relatively complex [21,22]. Besides, traditional RLNC comes with a sacrifice in service delay because if the users are not able to collect a full size of the encoding packets, the useful information cannot be recovered under the wireless fading channel [23]. Compared with the conventional relay system, PNC can double the throughput of a two-way relay channel (TWRC) by reducing the time slots for the exchange of one packet from four to two [24]. It has been a common belief that PNC requires tight synchronization [25], which is difficult to achieve in UAV networks. Complex field network coding (CFNC), as a generalized version of RLNC, is simple to implement and can facilitate the transmission of 1/2 symbol per source per channel use for multi-source cooperative relay networks [26]. Furthermore, the symbol-level synchronization of CFNC is more convenient to attain than bit-level synchronization [27]. In view of the above advantages, the CFNC is introduced to UAV cooperative surveillance networks in this paper.

**Figure 2:**
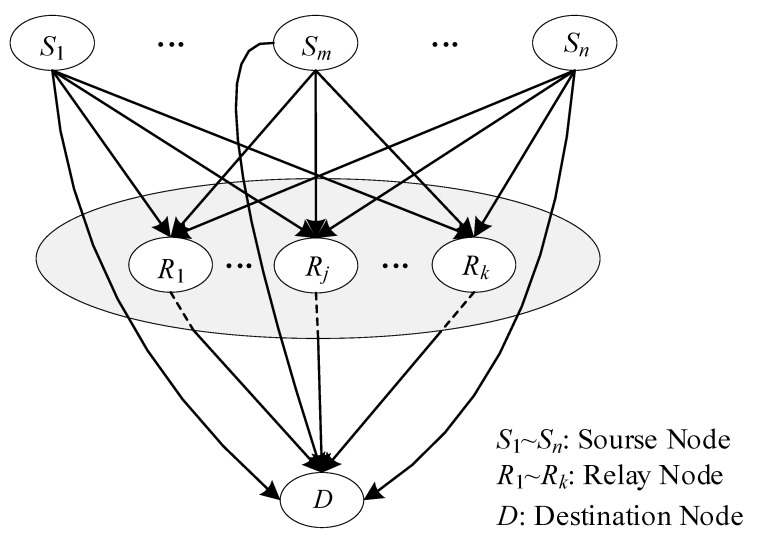
The conventional topology structure of network coding.

**Figure 3:**
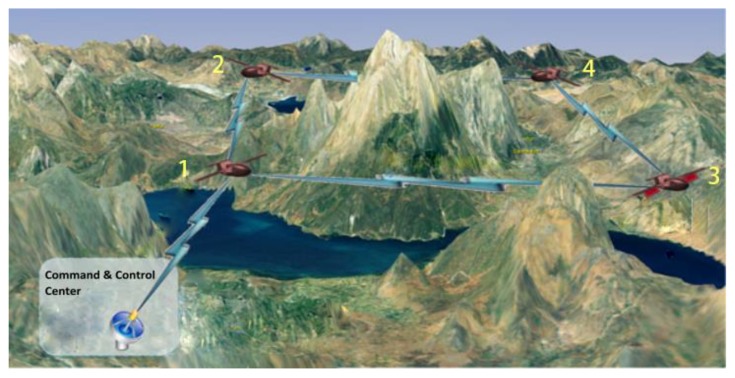
An example of an unmanned aerial vehicle (UAV) cooperative surveillance network being applied in a mountainous area.

The topology structure of NC is also multi-source multi-relay single-destination, as shown in Figure 2 [28]. In the structure, each source node simultaneously connects all relay nodes and the destination node. Moreover, all relay nodes are connected with the destination node. Any source node or relay node links the same numbers of edges, so this structure is called the regular structure by this paper. However, the regular structure is inapplicable to a dynamic time-varying UAV network for two main reasons. One is that not all source drones are always connected with the command and control center (destination node) when the distance between them is beyond communication range, typically for the purpose of expanding the surveillance range or because some obstacles are between them, as illustrated in Figure 3 [29]. It can be seen from Figure 3 that number 4 drone does not have a direct communication link to the command and control center because of a mountain barrier. The other reason for inapplicability is that every source drone cannot be always connected with all relay nodes due to its own mobility or some obstacles between them. In practical applications, any source drone should not always connect with all relay nodes and destination node simultaneously, and the corresponding structure is described as an irregular structure. According to whether there is a direct communication link between any source drone and the command and control center, the information transfer mechanism in downlink is set to one of two modes, either mixed or relay transmission. The specific meaning of mixed and relay transmissions will be expanded upon in Section 2.

The rest of this paper is organized as follows: Section 2 presents two irregular topology structures for a CFNC-based network according to the mixed and relay modes. For the different NC schemes, both throughput performance evaluation and the encoding/decoding derivation of CFNC in the two modes are provided by Section 3. Section 4 mainly analyzes the reliability of CFNC combined with the two proposed topology structures over an additive white Gaussian noise (AWGN) channel. Finally, we conclude the paper in Section 5.

## 2. Design of the Topology Structure

In order to enlarge the coverage area, a UAV cooperative network for surveillance purposes has to employ some drones as relay nodes to transmit messages. A very common topology structure in UAV cooperative networks is multiple surveillance drones, multiple relay drones, and a single command and control center. As shown in Figure 2, a conventional topology structure of NC consists of some source and relay nodes, as well as a destination node. If the source nodes, relay nodes, and the destination node are considered as surveillance drones, relay drones, and the command and control center, respectively, the topology structure of NC is similar to that of the surveillance network. Theoretically, the structure of the former could be applied to the latter.

The prominent feature of a NC structure is that each source node is always connected with all relay drones and the command and control center on the ground. However, this feature is not suitable for the changing dynamics of UAV cooperative networks. On the one hand, some source drones cannot deliver messages to the destination directly because the distance between them exceeds the maximum communication range or because direct communication is blocked by certain obstacles, such as mountains or buildings. On the other hand, it is unreasonable to expect every source drone to connect with all relay drones as obstacle blocking is likely to appear, or the distance among them may be beyond their own individual communication range. From this point of view, the topology structure of NC needs to be appropriately revised before application.

For the multi-source multi-relay single-destination structure expressed as *Ns*-*Nr*-1, the edges among different types of nodes are the most important factor influencing the total performance of the UAV cooperative surveillance network when the number of source drones (*Ns*) and relay drones (*Nr*) is fixed. The edge refers to a direct communication link between any two different types of nodes in this paper. These edges are divided into three groups, namely, edges between source nodes and the destination node, edges between source nodes and relay nodes, and edges between relay nodes and the destination node. The *Ns*-*Nr*-1 structure is made up of three types of node and a certain number of edges, so we can consider the structure as a special triple bipartite graph. Based on the characteristics of the bipartite graph, three group edges can be represented by different matrices. A row matrix, M, is introduced to express the edges between the source nodes and the destination node. If the *i*th element of $m_{i}$ in the matrix is equal to ‘1’, this indicates that the *i*th source node $S_{i}$ can deliver messages to the destination node D directly without a relay. Additionally, if $m_{i}=0$ this means there is no direct communication link between $S_{i}$ and D. Likewise, matrix G is employed here to represent the edges between the source nodes and relay nodes, and the rows and columns of this matrix indicate the relay and source nodes, respectively. If the element $G_{ij}$ in the matrix is ‘1’, this means that there is a direct communication link between the source node $S_{j}$ and the relay node $R_{i}$. Here, if $G_{ij}=0$ this represents that $S_{j}$ cannot send messages to $R_{i}$. For convenience, we assume that all relay nodes are always connected to the destination node, which means the edges between them can be expressed as an identity row matrix.

For the conventional topology structure of NC, as illustrated in Figure 2, $M_{1\times n}$ and $G_{k\times n}$ are both identity matrices, which is why we call the structure a regular structure. Through the above analysis, we may draw a conclusion that the regular structure of NC is not suitable for UAV cooperative surveillance networks, that is to say that all elements in $M_{1\times n}$ and $G_{k\times n}$ cannot always be equal to ‘1’. The number of edges is variable, even if *Ns* and *Nr* are constant, which leads to the diversity in structure. Similar to the characteristics of a check matrix in low-density parity-check (LDPC) codes, the density of ‘1’ in the both matrices is uncertain. The uncertainty results in a large number of irregular structures, even if the values of *Ns* and *Nr* are small. According to whether there is a direct communication link between any source drone and the command and control center, the information transfer mechanism at the downlink is set one of two modes, either mixed or relay transmission. In the first mode, the information is transmitted from all source drones to the destination by at least a direct link and multi-relay forwarding, which indicates that M is a non-zero matrix. In the other mode, all the source drones deliver messages to relay nodes within their communication range, that is to say, no direct communication link between the source nodes and the destination can be utilized, which means that M is a zero matrix.

**Figure 4:**
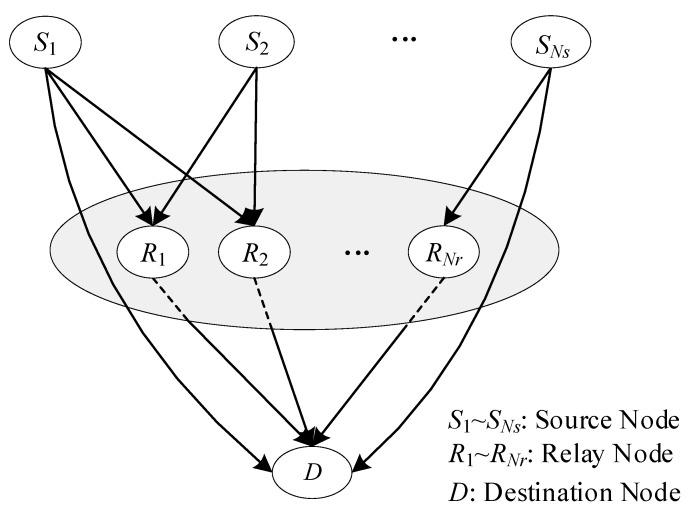
The irregular topology structure for the mixed transmission mode.

**Figure 5:**
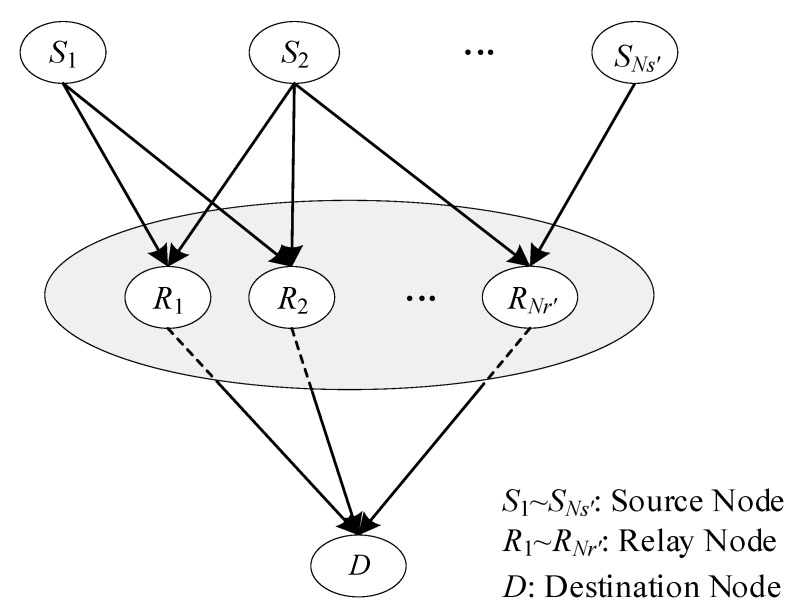
The irregular topology structure for the relay transmission mode.

Based on the two modes, two corresponding irregular topology structures for a CFNC-based network are proposed and Figure 4 and Figure 5 will serve as an example. The matrix M is set to ${\left[1\;0\;\cdots \;1\right]}_{1\times Ns}$ and ${\left[0\;0\;\cdots \;0\right]}_{1\times Ns’}$ in the mixed and relay modes, respectively, and the matrix G in the two modes is represented as follows, respectively:

(1) $${\left[\begin{array}{llll} 1 & 1 & \cdots & 0\\ 1 & 1 & \cdots & 0\\ \vdots & \vdots & \cdots & \vdots \\ 0 & 0 & \cdots & 1 \end{array}\right]}_{Nr\times Ns}$$

(2) $${\left[\begin{array}{llll} 1 & 1 & \cdots & 0\\ 1 & 1 & \cdots & 0\\ \vdots & \vdots & \cdots & \vdots \\ 0 & 1 & \cdots & 1 \end{array}\right]}_{Nr’\times Ns’}$$

The process of information transmission in the two topology structures is quite different. For the mixed mode, source drones will transmit information to the destination node via available direct links and the relay nodes within communication range simultaneously in the first time slot. In the second time slot, the relay nodes deliver the demodulated information to the destination node. In the second mode, all the source drones will transmit information to the relay nodes within communication range in the first time slot, then the relay drones deliver the demodulated information to the destination node in the second time slot.

## 3. Network Coding

In traditional relay communications, each source node takes advantage of a different time slot to transmit information, and each relay node also successively uses a different time slot to deliver information, which will result in poor real-time performance for information transmission [30]. Network coding can greatly reduce time slots, and the excellent characteristics of this suggest network coding has a very promising future in wireless multicast networks [31,32]. The classification of network coding, different network coding performance evaluations, and the encoding and decoding derivation of CFNC in the two modes are provided by Section 3.

### 3.1. The Classification of Network Coding

Based on the arithmetic mode, network coding can be divided into several categories, such as the binary field, the Galois field, complex field, and so on. The application of network coding in UAV cluster must consider the characteristics of UAV communication. With the application of new mission payloads, such as large-area and high-resolution digital aerial cameras, synthetic aperture radars, infrared imagers, etc., the information quantity detected by drones is growing exponentially. Saving on the return time of reconnaissance information implies a decrease in discovery probability. Next, we investigate which network coding scheme has the best real-time performance.

**Figure 6:**
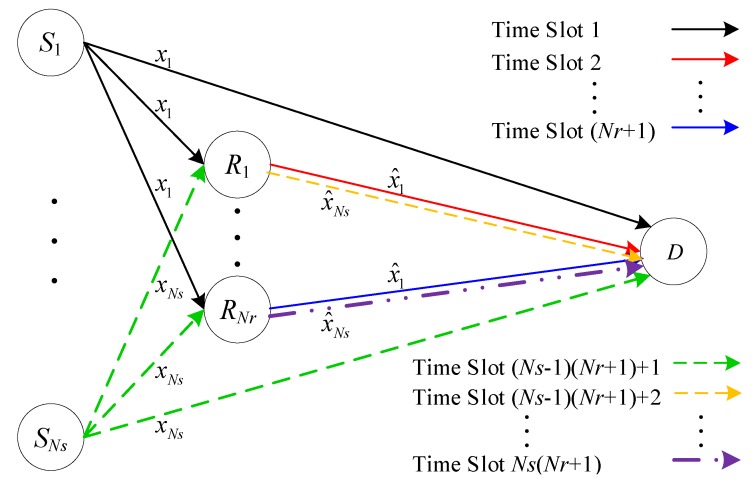
Traditional relay.

In general, network coding designs are based on the Galois field, which implements bit level operations. This coding scheme can improve throughput to some extent, but the advantage is diminished with an increasing number of source and relay nodes. A *Ns*-source *Nr*-relay single-destination structure with traditional network coding is depicted in Figure 6. Assuming that each node is equipped with an antenna, *Ns* sources ($S_{1},\cdots ,S_{Ns}$) transmit information to the destination (D) directly and via the relays ($R_{1},R_{2},\cdots ,R_{Nr}$). To avoid interference, sources $S_{1},\cdots ,S_{Ns}$, in the traditional relay format, transmit over orthogonal channels, e.g., via time division multiple access (TDMA) [27]. To start with, source $S_{1}$ transmits information symbols $x_{1}$ to $R_{1},R_{2},\cdots ,R_{Nr}$ and D simultaneously during channel use (CU) 1. Then, the relay $R_{1}$ forwards ${\hat{x}}_{1}$ to D in CU 2, and ${\hat{x}}_{1}$ is the decoding output of $R_{1}$ according to $x_{1}$. From CU 3 to CU (*Nr*+1), the $R_{2},\cdots ,R_{Nr}$ relays send ${\hat{x}}_{1}$ to D successively. The information symbol $x_{1}$ takes (*Nr*+1) CU from source $S_{1}$ to the destination D through relays $R_{1},R_{2},\cdots ,R_{Nr}$. For the information symbol sequence $\left\{x_{1},x_{2},\cdots ,x_{Ns}\right\}$, a total of *Ns*(*Nr*+1) channel uses are needed to deliver *Ns* symbols with *Ns* sources, and the throughput of this scheme is 1/(*Ns*(*Nr*+1)) symbol per source per channel use (sym/S/CU).

**Figure 7:**
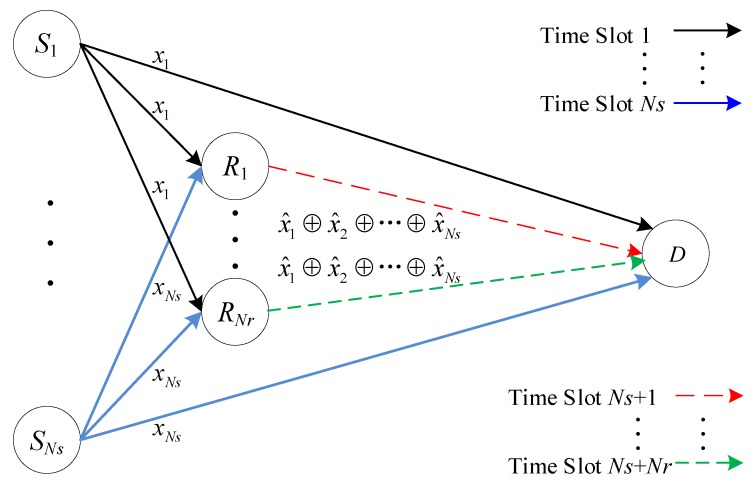
Relay with Galois field network coding (GFNC).

The relay scheme based on Galois field network coding (GFNC) is depicted in Figure 7. In CU 1, source $S_{1}$ transmits information symbol $x_{1}$ to both $R_{1},R_{2},\cdots ,R_{Nr}$ and D, the same as in a traditional relay. From CU 2 to CU *Ns*, information symbols $x_{2},\cdots ,x_{Ns}$ are sent to $R_{1},R_{2},\cdots ,R_{Nr}$ and D successively. $R_{1}$ forwards the Galois field coded symbol ${\hat{x}}_{1}⊕{\hat{x}}_{2}⊕\ldots ⊕{\hat{x}}_{Ns}$ to D in CU (*Ns*+1), where ⊕ denotes a bitwise exclusive XOR operation. Likewise, $R_{Nr}$ forwards the Galois field coded symbol ${\hat{x}}_{1}⊕{\hat{x}}_{2}⊕\ldots ⊕{\hat{x}}_{Ns}$ to D in CU (*Ns*+*Nr*). From the above analysis, we can deduce that (*Ns*+*Nr*) channel uses are needed for information symbol sequence $\left\{x_{1},x_{2},\cdots ,x_{Ns}\right\}$ transmission from *Ns* sources to D. Thus, the throughput of a GFNC-based relay is 1/(*Ns*+*Nr*) sym/S/CU.

**Figure 8:**
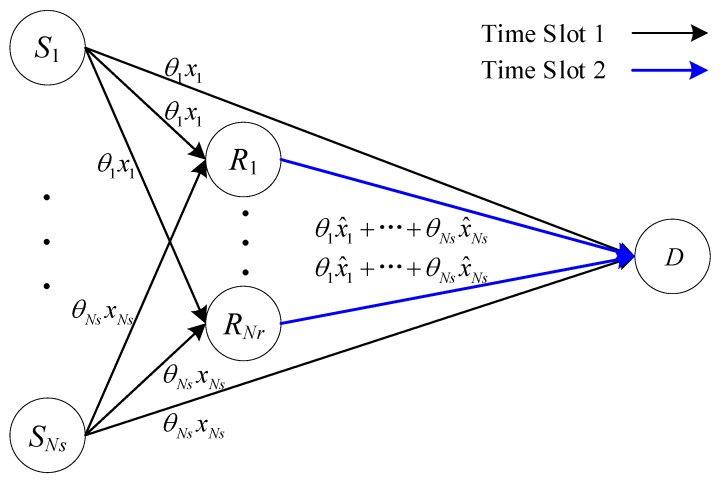
Relay with complex field network coding (CFNC).

For improving the real-time performance, a CFNC is introduced in this paper. As illustrated in Figure 8, before transmission in time slot 1, the source information $x_{i}$ from $S_{i}$ is multiplied by $\theta_{i}$, which is the *i*th element of $\theta_{S}^{T}=\left[\theta_{1},\theta_{2},\cdots ,\theta_{Ns}\right]$. We assume that $\theta_{S}^{T}$ is available at every node in the network. The choice for a diversity maximizing $\theta_{S}^{T}$ value is not unique but is available for any *Ns*. Among the different (parametric/non-parametric) choices for $\theta_{S}^{T}$, [28] takes it to be any row of the Vandermonde matrix, i.e.:

(3) $$\theta ={\left[\begin{array}{llll} 1 & \delta_{1} & \cdots & \delta_{1}^{Ns-1}\\ 1 & \delta_{2} & \cdots & \delta_{2}^{Ns-1}\\ \vdots & \;\vdots & \cdots & \vdots \\ 1 & \delta_{Ns} & \cdots & \delta_{Ns}^{Ns-1} \end{array}\right]}_{Ns\times Ns}$$

where the so-called generators, ${\left\{\delta_{n}\right\}}_{n=1}^{Ns}$, have a unit modulus in complex field C. Relays $R_{1},\cdots ,R_{Nr}$ simultaneously receive information symbols $\theta_{1}x_{1},\cdots ,\theta_{Ns}x_{Ns}$, transmitted by $S_{1},\cdots ,S_{Ns}$ in CU 1, and the agreed coefficients $\theta_{1},\cdots ,\theta_{Ns}$ drawn from C will be specified later. After detecting $x_{1},\cdots ,x_{Ns}$ as ${\hat{x}}_{1},\cdots ,{\hat{x}}_{Ns}$, $R_{1},\cdots ,R_{Nr}$ forwards $\theta_{1}{\hat{x}}_{1}+\ldots +\theta_{Ns}{\hat{x}}_{Ns}$ to D in CU 2. Therefore, the throughput of CFNC is 1/2 sym/S/CU. The throughput comparison of the above three schemes is listed in Table 1.

As can be seen from Table 1, GFNC is superior to traditional coding in terms of throughput, and the advantage gradually decreases with the increasing number of source and relay nodes, but CFNC can naturally avoid such a problem. The unique coding method employed by CFNC makes the throughput increase to 1/2 sym/S/CU, which is beneficial to improving the real-time performance. Moreover, the XOR operation is usually adopted by the GFNC, which will cause one-to-one mapping to be impossible between the source information and the received information. By contrast, the received information $\hat{u}$ ($\hat{u}=\theta_{1}{\hat{x}}_{1}+\cdots +\theta_{Ns}{\hat{x}}_{Ns}$) and information symbol sequence $\left\{x_{1},\cdots ,x_{Ns}\right\}$ easily satisfy one-to-one mapping, unless $x_{1}=x_{2}=\cdots =x_{Ns}$. Meanwhile, the mapping offers a method to detect ${\hat{x}}_{1},\cdots ,{\hat{x}}_{Ns}$ through the received information $\hat{u}$.

### 3.2. Information Transmission Based on Complex Field Network Coding (CFNC) in Mixed Mode

**Figure 9:**
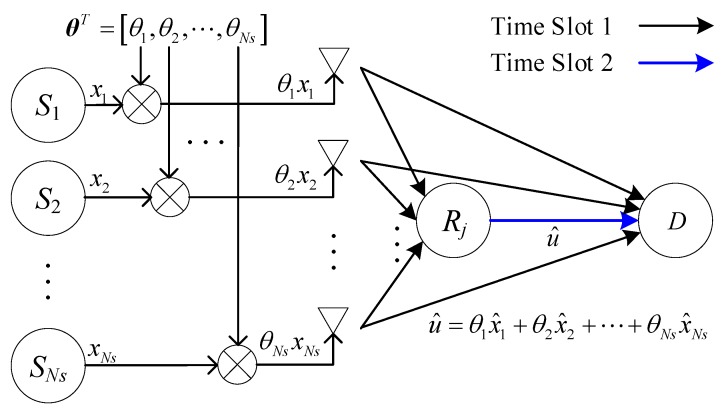
*Ns*-source setup with CFNC in the mixed mode.

Based on the theoretical analysis in the previous section, we have deduced that the CFNC obtains overwhelming superiority over other network coding schemes in terms of throughput when the source and relay nodes are of large quantities. Next, the information transmissions based on CFNC applied to the proposed topology structures is derived for the mixed and relay modes, respectively. According to the irregular topology structure *Ns*-*Nr*-1 for the mixed mode, as shown in Figure 4, the information symbol transmission based on CFNC merely involves two channel uses. The received symbols at $R_{j}$ and D after CU 1 are given as follows (see Figure 9):

(4) $$\begin{array}{ll} y_{SR_{j}}\left(t\right) & =h_{S_{1}R_{j}}\theta_{1}x_{1}(t)+\cdots +h_{S_{Ns}R_{j}}\theta_{Ns}x_{Ns}(t)+n_{SR_{j}}\left(t\right)\\ & =\theta_{S}^{T}H_{SR_{j}}x\left(t\right)+n_{SR_{j}}\left(t\right) \end{array},$$

(5) $$\begin{array}{ll} y_{SD}\left(t\right) & =h_{S_{1}D}\theta_{1}x_{1}(t)+\cdots +h_{S_{Ns}D}\theta_{Ns}x_{Ns}(t)+n_{SD}\left(t\right)\\ & =\theta_{S}^{T}H_{SD}x\left(t\right)+n_{SD}\left(t\right) \end{array},$$

where for each subscript duplet, $h_{ij}∼CN(0,\sigma_{ij}^{2})$ denotes the channel coefficient and $n_{ij}∼CN(0,N_{0})$ denotes the AWGN term. The instantaneous and average signal-to-noise ratios (SNRs) are given respectively by ${ϒ}_{ij}={\left|h_{ij}\right|}^{2}\bar{ϒ}$ and ${\bar{ϒ}}_{ij}=\sigma_{ij}^{2}\bar{ϒ}$, where $\bar{ϒ}=P_{x}/N_{0}$ and $P_{x}$ denote the average transmission power of source symbol x, which is assumed to be drawn from a finite alphabet $A_{x}$ with cardinality $\left|A_{x}\right|$ [27]. Here, $H_{SR_{j}}=\mathrm{diag}(h_{S_{1}R_{j}},h_{S_{2}R_{j}},\cdots ,h_{S_{Ns}R_{j}})$, $H_{SD}=\mathrm{diag}(h_{S_{1}D},h_{S_{2}D},\cdots ,h_{S_{Ns}D})$, and information symbol vector $x(t)={\left[x_{1}(t),\cdots ,x_{Ns}(t)\right]}^{T}$, where t=1,⋯,Nr and j=1,⋯,Nr.

The design of $\theta_{S}^{T}$ in Equations (4) and (5) is critical to CFNC. The design relates the linear complex field (LCF) encoder given in [33] for multiple input multiple output (MIMO) systems. Based on the concept of Euler numbers and their properties, two systematic designs of these generators are provided in [34]: $\delta_{n}=e^{j\pi (4n-1)/2Ns}$ if $Ns=2^{k}$ and $\delta_{n}=e^{j\pi (6n-1)/3Ns}$ if $Ns=3\times 2^{k}$, where n indicates the *n*th row of Vandermonde matrix. In other words, $\theta_{i}=e^{j\pi (4n-1)(i-1)/2Ns}$ if $Ns=2^{k}$ and $\theta_{i}=e^{j\pi (6n-1)(i-1)/3Ns}$ if $Ns=3\times 2^{k}$, where i=1,⋯,Ns. However, the similarities with MIMO-LCF designs stop here. Notice that the coded symbol $u=\theta_{1}x_{1}+\cdots +\theta_{Ns}x_{Ns}$ in CFNC is transmitted through different nodes (sources) in the network simultaneously, instead of through multiple co-located antennas on one terminal [33]. Therefore, a normalizing factor, as in ([34], Eq. (3.68)), to meet the power constraint on one node is not necessary here [28].

After Nr relay channels, the maximum likelihood (ML) of detection at relay $R_{j}$ is given as follows:

(6) $${\hat{x}}_{j}(t)=\arg\underset{x\left(t\right)}{\min}\left\|y_{{\mathrm{SR}}_{j}}\left(t\right)-\theta_{S}^{T}H_{SR_{j}}x\left(t\right)\right\|,$$

The relaying node $R_{j}$ re-encodes the demodulation results then sends it to the target node. The input/output (I/O) relationship in CU 2 is expressed as follows:

(7) $$y_{R_{j}D}\left(t\right)=\sqrt{\alpha_{j}}h_{R_{j}D}\theta_{R}^{T}{\hat{x}}_{j}+n_{R_{j}D},\;j=1,\cdots ,Nr,$$

where ${\hat{x}}_{j}={\left[{\hat{x}}_{j}^{T}(1),\cdots ,{\hat{x}}_{j}^{T}(Nr)\right]}^{T}$, $\alpha_{j}$ represents a link-adaptive scalar which controls the transmission power at $R_{j}$, $\theta_{R}$ is an NsNr×1 vector designed as the above, i.e., $\theta_{R}^{T}=\left[\theta_{1}^{’},\theta_{2}^{’},\cdots ,\theta_{Ns\times Nr}^{’}\right]$. For $Nr\times Ns=2^{k}$, the entries of $\theta_{R}$ are given by $\theta_{i}^{’}=e^{j\pi \left(4n-1\right)\left(i-1\right)/\left(2Ns\times Nr\right)}$ and i=1,2,⋯,Ns×Nr, and for $Nr\times Ns=3\times 2^{k}$, $\theta_{i}^{’}=e^{j\pi \left(6n-1\right)\left(i-1\right)/\left(3Ns\times Nr\right)}$ for any n=1,2,⋯,Ns×Nr.

The symbol rate is 1/2 sym/S/CU, because *Ns* sources transmit *Ns* signals over 2 channels. After passing through 2 channels, the ML detection result at D is given as follows:

(8) $${\hat{x}}_{D}=\arg\underset{x’}{\min}\{\sum_{t=1}^{Nr}{\left\|y_{SD}(t)-\theta_{S}^{T}H_{SD}x(t)\right\|}^{2}\;+\sum_{j=1}^{Nr}{\left\|y_{R_{j}D}(t)-\sqrt{\alpha_{j}}h_{R_{j}D}\theta_{R}^{T}x’\right\|}^{2}\},$$

where $x’={\left[x^{T}(1),\cdots ,x^{T}(Nr)\right]}^{T}$.

### 3.3. Information Transmission Based on CFNC in Relay Mode

**Figure 10:**
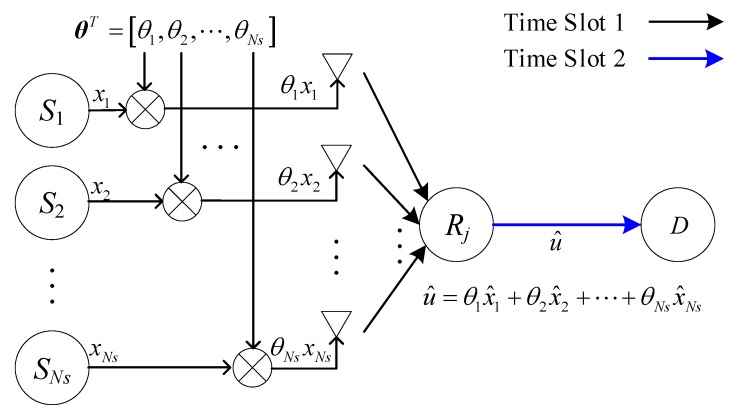
*Ns*-source setup with CFNC in the relay mode.

There are no any direct communication links between the source drones and the command and control center when the source drones move beyond their communication range or the links among them are totally blocked. In such a situation, the conventional topology structure of NC exhibited in Figure 4 is inapplicable for such an application. Thus, an irregular topology structure in the relay mode is proposed by this paper, depicted in Figure 5. The received symbols at $R_{j}$ after CU 1 (see Figure 10) are the same as in Section 3.2, i.e., $y_{SR_{j}}(t)=\theta_{S}^{T}H_{SR_{j}}x(t)+n_{SR_{j}}(t)$.

After *Nr* channel uses, relay $R_{j}$ detects ${\hat{x}}_{j}(t)=\arg\underset{x\left(t\right)}{\min}$ $\left\|y_{SR_{j}}\left(t\right)-\theta_{S}^{T}H_{SR_{j}}x\left(t\right)\right\|$ and forwards this demodulated symbol with scaling coefficient $\alpha_{j}$ in next CU. The I/O relationship is $y_{R_{j}D}\left(t\right)=\sqrt{\alpha_{j}}h_{R_{j}D}\theta_{R}^{T}{\hat{x}}_{j}+n_{R_{j}D}$, where j=1,2,⋯,Nr, where $\theta_{R}$ is the NsNr×1 vector designed in Section 3.2.

Since *Nr* symbols are transmitted per source over 2*Nr* channel uses, the symbol rate is clearly 1/2 sym/S/CU. After passing through 2 channels, the ML detection result at D is given as follows:

(9) $${\hat{x}}_{D}=\arg\underset{x’}{\min}\left\{\sum_{t=1}^{Nr}\sum_{j=1}^{Nr}{\left\|y_{R_{j}D}(t)-\sqrt{\alpha_{j}}h_{R_{j}D}\theta_{R}^{T}x’\right\|}^{2}\right\},$$

where the calculation method of $\theta_{R}$ is referred to the previous section.

## 4. Simulation Results and Analysis

### 4.1. Topology Structure Performance Evaluation

The throughput performance of CFNC based on an irregular topology structure in the mixed mode has been assessed in Section 3.1. Compared with CFNC, based on the conventional topology structure, the reliability of CFNC applied in the two proposed topology structures over an AWGN channel has been evaluated by Monte Carlo simulations using MATLAB. In this section, we mainly investigate the influence of the source and relay node numbers to the symbol error probability (SEP) of the two proposed structures. In all simulations, the frame length of information transmitted by each source node was 1000 bits, and the bits in the same position of every information frame constituted a single symbol, i.e., a symbol contained *Ns* bits. The frame number of each source node was fixed at 1500.

**Figure 11:**
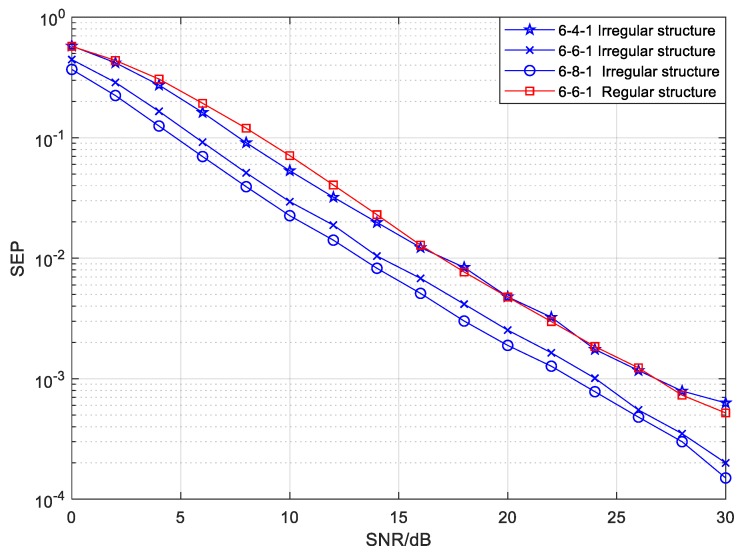
The symbol error probability (SEP) of the mixed mode with different numbers of relays in a 6-*Nr*-1 CFNC-based structure.

**Figure 12:**
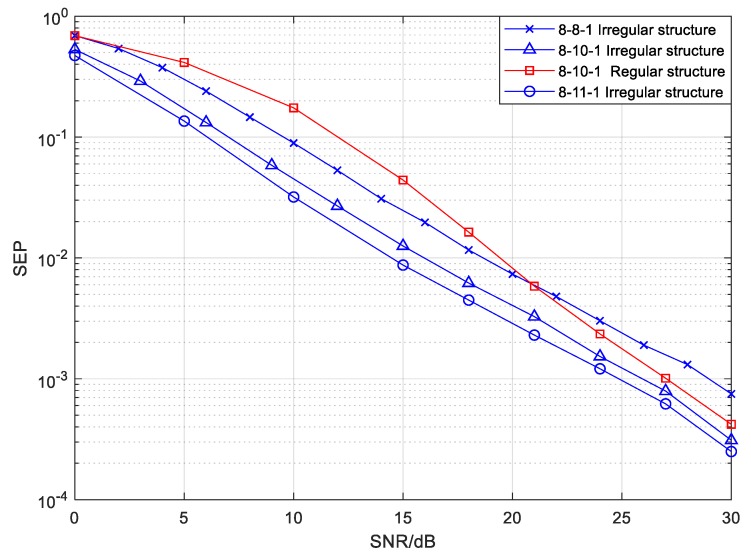
The SEP of the mixed mode with different numbers of relays in a 8-*Nr*-1 CFNC-based structure.

We investigated the mixed mode reliability of the proposed irregular topology structure with different numbers of source and relay drones compared to the regular structure. Figure 11 and Figure 12 show the SEP performance of the mixed mode with different numbers of relays in the 6-*Nr*-1 and 8-*Nr*-1 structures, respectively. The edge parameters of the 6-*Nr*-1 and 8-*Nr*-1 structures in the mixed mode are exhibited in Table 2 and Table 3 separately, and the other simulation parameters were the same as mentioned above if no special indication is otherwise given. It can be seen from Figure 11 that the SEP performance of the mixed mode increases better with the increasing number of relay drones when the number of source drones is fixed at 6. This is due to the higher diversity gains originating from the increasing number of relay nodes. However, the space for SEP improvement gradually diminishes when increasing the relay drone number when *Nr* is larger than 6. In order to reduce the complexity and cost of UAV cooperative networks, we selected the number of relay drones as 6 for the 6-*Nr*-1 structure. Compared with the regular 6-6-1 structure, the proposed 6-6-1 structure earns gains of at least 3 dB in the region of SEP = $10^{-3}$, that is to say, the irregular structure can remarkably improve reliability over the regular structure under the same parameters.

For the 8-*Nr*-1 irregular structure in the mixed mode, the simulation results of the SEP performance shown in Figure 12 are very similar to those in Figure 11. As we see from Figure 12, the SEP decreased with an increasing number of relay drones when the number of source drones was set at 8. It is noteworthy that the improvement on SEP is smaller when the number of relay drones is greater than 10. Too many relay nodes will increase the complexity and cost of a UAV cluster. In view of the reasons given above, the number of relay nodes was selected as 10 for the 8-*Nr*-1 structure. In addition, the reliability of the irregular 8-10-1 structure was superior to that of the regular structure under the same simulation parameters. Through the above analysis, we can deduce that the proposed irregular topology structure in the mixed mode has certain advantages in terms of the reliability when compared with the regular structure under the same conditions.

**Figure 13:**
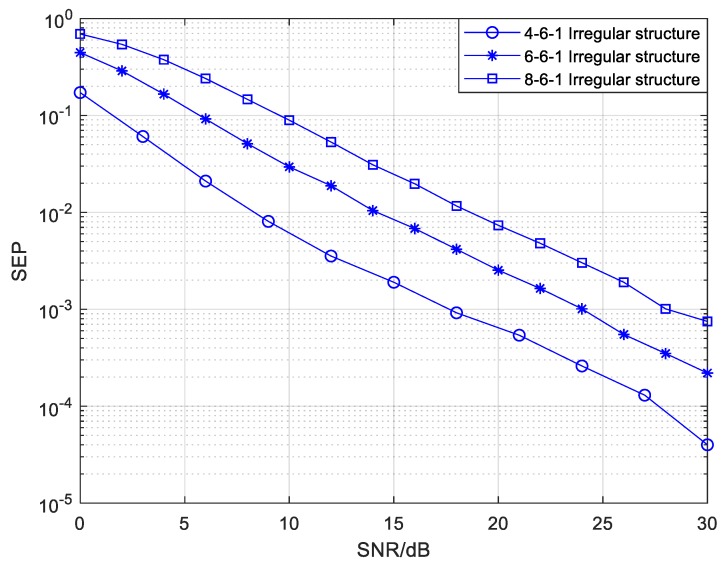
The SEP of the mixed mode with different numbers of sources in the *Ns*-6-1 CFNC-based structure.

The effect of the source drone number on the SEP performance of the irregular structure in the mixed mode is illustrated in Figure 13. More details about the edge setting in the *Ns*-6-1 structure are exhibited in Table 4. We can observe from Figure 13 that the SEP performance worsens with an increasing number of source drones when the number of relay drones is fixed at 6. For a single relay node, the more information it receives from the connected source drones, the worse the SEP performance is. The interference among different messages will be intensified when a relay node processes or forwards information, which results in poor SEP performance.

**Figure 14:**
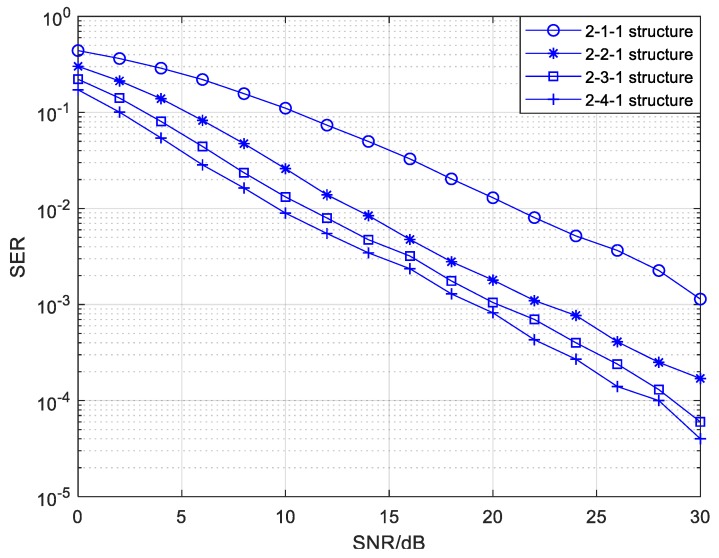
The SEP of the relay mode with different numbers of relays in the 2-*Nr*-1 CFNC-based structure.

In the relay mode, there is no any connection between the source drones and the command and control center, which indicates that M is a zero-row matrix. Figure 14 shows the SEP performance of the relay mode with different numbers of relays in the 2-*Nr*-1 irregular topology structure. More details about the edge setting in the 2-*Nr*-1 structure are exhibited in Table 5. As we see from Figure 14, the SEP performance is gradually improved with an increasing number of relay drones when the number of source drones is fixed at 2. This is because more relay nodes bring more diversity gains, which leads to a better reliability. It is noteworthy that the room for improvement on the SEP performance is limited when the number of relay nodes is larger than a certain value. Moreover, the increasing number of relay nodes will impose a relatively high implementation complexity and cost for cooperative UAV networks. Therefore, the selection of the relay number should take into account reliability, network complexity, system cost, and so on.

**Figure 15:**
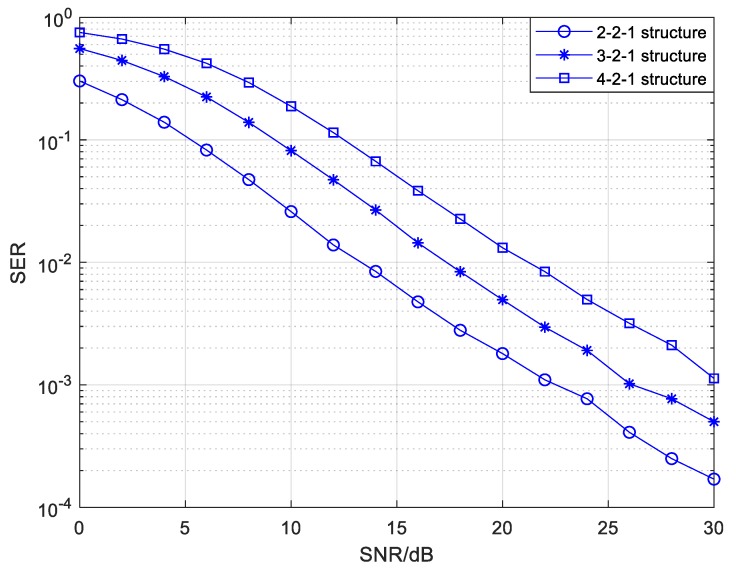
The SEP of the relay mode with different numbers of sources in the *Ns*-2-1 CFNC-based structure.

The effect of the source drone number on the SEP performance of the irregular structure in the relay mode is illustrated in Figure 15. The detailed edge parameters in the *Ns*-2-1 structure are exhibited in Table 6. It can observed from Figure 15 that the SEP performance gets worse with an increasing number of source drones. The reason for this is similar to that of the mixed mode. The greater the number of source drones a single relay node links, the more messages it receives. The mutual interference among messages goes against data processing and forwarding, which leads to a considerable decline in reliability.

### 4.2. The Combination of CFNC and Conventional Unmanned Aerial Vehicle (UAV) Datalink

**Figure 16:**
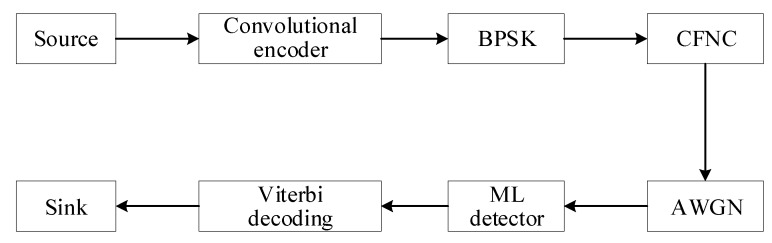
The transmission scheme of coded binary phase shift keying complex field network coding (CC-BPSK-CFNC).

Through the above analysis, we can deduce that the CFNC applied in the proposed irregular structures based on the two transmission modes has a distinct advantage in terms of the reliability and throughput found. Next we discuss the performance of CFNC combined with a UAV datalink signal system and convolutional coded binary phase shift keying (CC-BPSK) modulation, which is a common transmission scheme used in existing UAV datalinks. Figure 16 shows a block diagram of CC-BPSK combined with CFNC (abbreviated as CC-BPSK-CFNC). In this system, the simulation parameters were set as follows: The structure of convolutional code was (2, 1, 3), i.e., one information bit was encoded into a 2-bit codeword each time (code rate was 1/2), and the constraint length was 3; the generator matrix was [1 1 1; 1 0 1]; and the Viterbi algorithm was adopted for decoding. The irregular topology structure of the CFNC in the two modes was chosen as 8-8-1, and the edge parameters in the structure are shown in Table 3.

**Figure 17:**
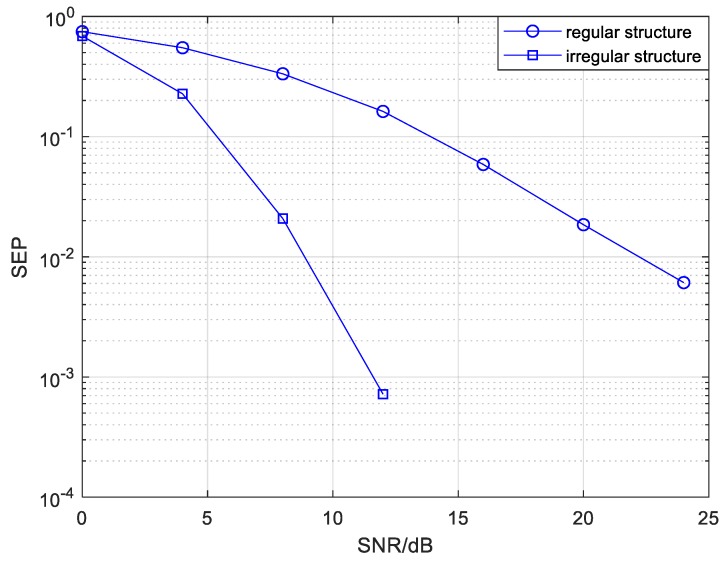
The SEP comparison of CC-BPSK-CFNC based on the mixed mode in regular and irregular structures.

**Figure 18:**
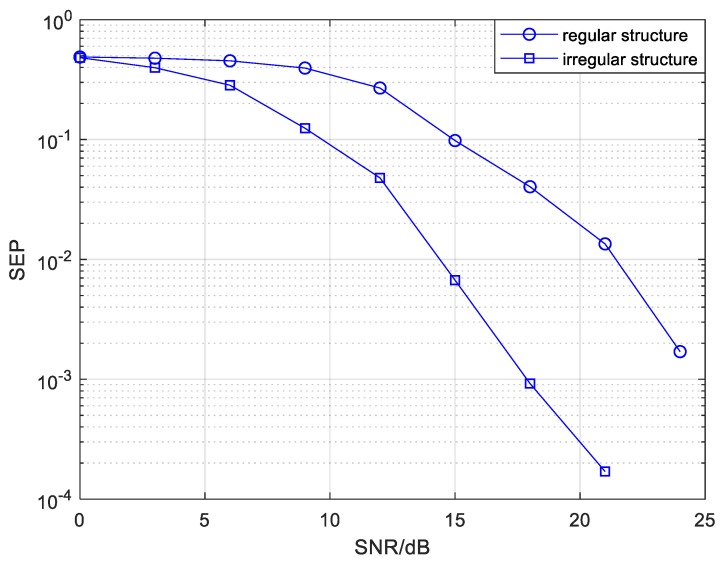
The SEP comparison of CC-BPSK-CFNC based on the relay mode in regular and irregular structures.

The SEP comparison of CC-BPSK-CFNC, based on the mixed mode in regular and irregular structures, is illustrated in Figure 17. As shown in Figure 17, a SEP value of $10^{-4}$ is attainable for CC-BPSK-CFNC in the irregular structure at a SNR of around 12 dB, whereas the equivalent SEP performance for CFNC based on the same structure without channel coding and modulation has a SNR of about 30 dB (as shown in Figure 12). Note that the reliability could be improved by invoking a few coded modulation techniques at the expense of rate loss. The transmission scheme, i.e., CC-BPSK-CFNC, in the irregular structure could obtain at least a 14 dB gain at the SEP of $5\times 10^{-3}$ compared with the scheme in the regular structure. The SEP comparison of the CC-BPSK-CFNC, based on relay mode in regular and irregular structures, is depicted in Figure 18. We can see that the SEP of $10^{-4}$ is attainable for CC-BPSK-CFNC in the irregular structure when the SNR is greater than 18 dB. Compared with the regular structure, the scheme based on the irregular one can earn at least a 6.5 dB gain with a SEP of $10^{-3}$.

## 5. Conclusions

Using multiple drones to form a collaborative network will become one of the main trends of UAV development in the future. The amount of interactive information among drones in such a collaborative network is expected to increase greatly. Complex field network coding (CFNC) is an effective method to improve network throughput and has been introduced to UAV cooperative surveillance networks in this paper, where the throughput was found to be as high as 1/2 sym/S/CU, which is superior to other network coding schemes. According to whether there is a direct communication link between any source drone and the destination, the information transfer mechanism at the downlink was set to one of two modes, either mixed or relay transmission, and two corresponding irregular topology structures for a CFNC-based network have been proposed, and the information transmissions based on CFNC in the mixed and relay modes were derived. The simulation results over an AWGN channel based on the MATLAB software show that the CFNC applied in the proposed irregular structures under the two transmission modes can remarkably improve reliability using the same parameters when compared with the regular structures. Moreover, the CFNC could easily be combined with the existing channel coding and modulations of UAVs datalinks, such as CC-BPSK, which continues to enhance the SEP performance to a great extent.

## Figures and Tables

**Table 1:** The throughput performance of various network coding schemes.

Network Coding Scheme	Number of Channels Occupied by the Source Nodes	Number of Channels Occupied by the Relaying Nodes	Throughput (Symbol/Source/Channel Use)
Traditional	*Ns*	*Ns×Nr*	1/(*Ns*(*Nr*+1))
GFNC	*Ns*	*Nr*	1/(*Ns*+*Nr*)
CFNC	1	1	1/2

**Table 2:** The edge parameters of the 6-*Nr*-1 structure in the mixed mode.

Structures	6-4-1	6-6-1	6-8-1
M	$\left[\begin{array}{cccccc} 1 & 1 & 1 & 0 & 1 & 1 \end{array}\right]$	$\left[\begin{array}{cccccc} 1 & 1 & 1 & 1 & 1 & 1 \end{array}\right]$	$\left[\begin{array}{cccccc} 1 & 1 & 1 & 1 & 1 & 1 \end{array}\right]$
G	$\left[\begin{array}{cccccc} 1 & 1 & 0 & 0 & 0 & 0\\ 1 & 1 & 1 & 1 & 0 & 0\\ 0 & 0 & 1 & 1 & 1 & 1\\ 0 & 0 & 0 & 0 & 1 & 1 \end{array}\right]$	$\left[\begin{array}{cccccc} 1 & 1 & 0 & 0 & 0 & 0\\ 1 & 1 & 1 & 0 & 0 & 0\\ 1 & 1 & 1 & 1 & 0 & 0\\ 0 & 1 & 1 & 1 & 1 & 0\\ 0 & 0 & 1 & 1 & 1 & 1\\ 0 & 0 & 0 & 1 & 1 & 1 \end{array}\right]$	$\left[\begin{array}{cccccc} 1 & 0 & 0 & 0 & 0 & 0\\ 1 & 1 & 0 & 0 & 0 & 0\\ 1 & 1 & 1 & 0 & 0 & 0\\ 1 & 1 & 1 & 1 & 0 & 0\\ 0 & 1 & 1 & 1 & 1 & 1\\ 0 & 0 & 1 & 1 & 1 & 1\\ 0 & 0 & 1 & 1 & 1 & 1\\ 0 & 0 & 0 & 0 & 1 & 1 \end{array}\right]$

**Table 3:** The edge parameters of the 8-*Nr*-1 structure in the mixed mode.

Structures	8-8-1	8-10-1	8-11-1
M	$\left[\begin{array}{c} \;1\;1\;1\;1\;1\;1\;1\;1\; \end{array}\right]$	$\left[\begin{array}{c} \;1\;1\;1\;1\;1\;1\;1\;0\; \end{array}\right]$	$\left[\begin{array}{c} \;1\;1\;1\;1\;1\;1\;1\;0\; \end{array}\right]$
G	$\left[\begin{array}{c} \;1\;1\;1\;1\;0\;0\;0\;0\;\\ \;1\;1\;1\;1\;0\;0\;0\;0\;\\ \;0\;1\;1\;1\;1\;0\;0\;0\;\\ \;0\;1\;1\;1\;1\;0\;0\;0\;\\ \;0\;0\;1\;1\;1\;1\;0\;0\;\\ \;0\;0\;0\;1\;1\;1\;1\;0\;\\ \;0\;0\;0\;1\;1\;1\;1\;0\;\\ \;0\;0\;0\;0\;1\;1\;1\;1\; \end{array}\right]$	$\left[\begin{array}{c} \;1\;1\;1\;1\;0\;0\;0\;0\\ \;1\;1\;1\;1\;0\;0\;0\;0\\ \;0\;1\;1\;1\;1\;0\;0\;0\;\\ \;0\;1\;1\;1\;1\;0\;0\;0\\ \;0\;0\;1\;1\;1\;1\;0\;0\\ \;0\;0\;1\;1\;1\;1\;0\;0\\ \;0\;0\;0\;1\;1\;1\;1\;0\\ \;0\;0\;0\;1\;1\;1\;1\;0\\ \;0\;0\;0\;0\;1\;1\;1\;1\\ \;0\;0\;0\;0\;1\;1\;1\;1 \end{array}\right]$	$\left[\begin{array}{l} \;1\;1\;1\;1\;0\;0\;0\;0\\ \;1\;1\;1\;1\;0\;0\;0\;0\\ \;0\;1\;1\;1\;1\;0\;0\;0\;\\ \;0\;1\;1\;1\;1\;0\;0\;0\\ \;0\;0\;1\;1\;1\;1\;0\;0\\ \;0\;0\;1\;1\;1\;1\;0\;0\\ \;0\;0\;0\;1\;1\;1\;1\;0\\ \;0\;0\;0\;1\;1\;1\;1\;0\\ \;0\;0\;0\;0\;1\;1\;1\;1\\ \;0\;0\;0\;0\;1\;1\;1\;1\\ \;0\;0\;0\;0\;0\;1\;1\;1 \end{array}\right]$

**Table 4:** The edge parameters of the *Ns*-*6*-1 structure in the mixed mode.

Structures	4-6-1	6-6-1	8-6-1
M	[ 1 1 1 1 ]	[ 1 1 1 1 1 1 ]	[ 1 1 1 1 1 1 1 0 ]
G	$\left[\begin{array}{l} \;1\;1\;0\;0\\ \;1\;1\;1\;0\\ \;1\;1\;1\;0\;\\ \;0\;1\;1\;1\;\\ \;0\;1\;1\;1\\ \;0\;0\;1\;1 \end{array}\right]$	$\left[\begin{array}{l} \;1\;1\;0\;0\;0\;0\\ \;1\;1\;1\;0\;0\;0\\ \;1\;1\;1\;1\;0\;0\\ \;0\;1\;1\;1\;1\;0\\ \;0\;0\;1\;1\;1\;1\;\\ \;0\;0\;0\;1\;1\;1\; \end{array}\right]$	$\left[\begin{array}{l} \;1\;1\;1\;1\;0\;0\;0\;0\\ \;1\;1\;1\;1\;0\;0\;0\;0\\ \;0\;1\;1\;1\;1\;0\;0\;0\\ \;0\;0\;1\;1\;1\;0\;0\;0\\ \;0\;0\;0\;1\;1\;1\;1\;0\;\\ \;0\;0\;0\;0\;1\;1\;1\;1\; \end{array}\right]$

**Table 5:** The edge parameters of the 2-*Nr*-1 structure in the relay mode.

Structures	2-1-1	2-2-1	2-3-1	2-4-1
G	[ 1 1 ]	$\left[\begin{array}{l} \;1\;1\;\\ \;1\;1\; \end{array}\right]$	$\left[\begin{array}{l} \;1\;0\\ \;1\;1\;\\ \;0\;1 \end{array}\right]$	$\left[\begin{array}{l} \;1\;0\\ \;1\;1\\ \;1\;1\;\\ \;0\;1 \end{array}\right]$

**Table 6:** The edge parameters of the *Ns*-2-1 structure in the relay mode.

Structures	2-2-1	3-2-1	4-2-1
G	$\left[\begin{array}{l} \;1\;1\;\\ \;1\;1 \end{array}\right]$	$\left[\begin{array}{l} \;1\;1\;0\\ \;0\;1\;1 \end{array}\right]$	$\left[\begin{array}{l} \;1\;1\;1\;0\\ \;0\;1\;1\;1 \end{array}\right]$

## Abbreviations

The following abbreviations are used in this manuscript:

UAV	Unmanned Aerial Vehicle
NC	Network Coding
GFNC	Galois Field Network Coding
RLNC	Random Linear Network Coding
PNC	Physical-layer Network Coding
CFNC	Complex Field Network Coding
AWGN	Additive White Gaussian Noise
MSMRSD	Multi-source multi-relay single-destination
LDPC	Low-density Parity-check
LCF	Linear Complex Field
TWRC	Two-way Relay Channel
TDMA	Time Division Multiple Access
CU	Channel Use
MIMO	Multiple Input Multiple Output
ML	Maximum Likelihood
CC	Convolutional Code
BPSK	Binary Phase Shift Keying
SEP	Symbol Error Probability
